# Changes in engagement in HIV prevention and care services among female sex workers during intensified community mobilization in 3 sites in Zimbabwe, 2011 to 2015

**DOI:** 10.1002/jia2.25138

**Published:** 2018-07-22

**Authors:** Tendayi Ndori‐Mharadze, Elizabeth Fearon, Joanna Busza, Jeffrey Dirawo, Sithembile Musemburi, Calum Davey, Xeno Acharya, Sibongile Mtetwa, James R Hargreaves, Frances Cowan

**Affiliations:** ^1^ Centre for Sexual Health HIV and AIDS Research (CeSHHAR Zimbabwe) Harare Zimbabwe; ^2^ Department of Public Health, Environments and Society London School of Hygiene and Tropical Medicine London UK; ^3^ Department of Population Health London School of Hygiene and Tropical Medicine London UK; ^4^ Epidemiology and Public Health Harvard TH Chan School of Public Health Boston MA; ^5^ Department of International Public Health Liverpool School of Tropical Medicine Liverpool UK

**Keywords:** Female sex workers, HIV, ART, peer education, community mobilization

## Abstract

**Introduction:**

‘Sisters with a Voice’, Zimbabwe's nationally scaled comprehensive programme for female sex workers (FSWs), intensified community mobilization activities in three sites to increase protective behaviours and utilization of clinical services. We compare indicators among FSWs at the beginning and after implementation.

**Methods:**

We used mixed methods to collect data at three sites: in‐depth interviews (n = 22) in 2015, routine clinical data from 2010 to 2015, and two respondent driven sampling surveys in 2011 and 2015, in which participants completed an interviewer‐administered questionnaire and provided a finger prick blood sample for HIV antibody testing. Estimates were weighted using RDS‐1 and estimate convergence assessed in both years. We assessed differences in six indicators between 2011 and 2015 using logistic regression adjusted for age, duration in sex work and education.

**Results:**

870 FSWs were recruited from the three sites in 2011 and 915 in 2015. Using logistic regression to adjust for socio‐demographic differences, we found higher estimates of the proportion of HIV‐positive FSWs and HIV‐positive FSWs who knew their status and reported being on ART in Mutare and Victoria Falls in 2015 compared to 2011. Reported condom use with clients did not differ by year; however, condom use with regular partners was higher in 2015 in Mutare and Hwange. Reported HIV testing in the last six months among HIV‐negative FSWs was higher in 2015 across sites: for instance, in Victoria Falls it was 13.4% (95% CI 8.7% to 19.9%) in 2011 and 80.8% (95% CI 74.0 to 87.7) in 2015. FSWs described positive perceptions of the Sisters programme, ease of engaging with health services, and improved solidarity among peers. Programme data showed increases in service use by 2015 across all sites.

**Conclusions:**

Improvements in key HIV care engagement indicators were observed among FSWs in two sites and in testing and prevention indicators across the three sites after implementation of an intensified community mobilization intervention. Engagement with services for FSWs is critical for countries to reach 90‐90‐90 targets.

## 1 Introduction

The World Health Organization and UNAIDS recommend that sex workers access comprehensive HIV prevention, testing, and treatment 1, 2, 3. Female sex workers (FSWs) have a high burden of HIV 4, and 15% of HIV infections in the general adult population globally are considered attributable to unsafe commercial sex 5. This proportion is likely to increase over time 6. Sex workers suffer criminalization 7, stigma, discrimination and violence in a number of settings, heightening their vulnerability to HIV 8, 9. Due to their social marginalization, sex workers often choose to access targeted, non‐judgmental, and tailored services to meet their needs 10, 11. Despite evidence of their effectiveness 12, 13, 14, targeted programmes for FSWs in most countries consist of small scattered projects, with limited scope and coverage 15, 16, 17, 18.

Evidence suggests that FSWs often have poor linkage to and retention in care 19 due to a range of factors including stigma and discrimination experienced within healthcare settings 20, 21, particularly if they are also living with HIV 22, 23. Peer to peer support, proximity to a targeted health centre, and “sex worker friendly” healthcare providers have been identified as key enablers of HIV testing and retention in care for HIV‐positive FSWs 19, 24.

The Zimbabwe National AIDS Strategic Plan 25 identifies FSWs as a key population at increased risk of HIV. Since 2009, the national “Sisters with a Voice” (Sisters) programme has provided free preventive and clinical services to FSWs supported by a network of trained peer educators. Services include syndromic management of sexually transmitted infections (STIs), provision of contraception, HIV testing and referral for HIV‐positive women to public sector clinics for antiretroviral therapy (ART). ART has been freely available through the public sector since 2004, but in 2011 there was evidence that FSWs were reluctant to attend public clinics due to encountering discriminatory attitudes 26, 27. In response, CeSHHAR Zimbabwe piloted an intensified community mobilization project in Mutare, Hwange and Victoria Falls between 2010 and 2015, based on evidence from India showing that community mobilization for sex workers can increase condom use and reduce HIV and STI rates among FSWs 28, 29, 30. More recent studies confirm that increased peer outreach is associated with FSWs’ improved health‐seeking and clinic attendance 31, 32.

We compare key indicators related to FSW health seeking behaviour in 2011 and 2015 in three sites. We explore whether observed differences might be linked to the delivery of intensified community mobilization.

## 2 Methods

We draw data from three sources to inform the conclusions; Respondent Driven Sampling (RDS) surveys conducted in 2011 33 and 2015, in‐depth interviews with FSWs selected as seeds of the 2015 RDS Survey, and review of programme data from the Sisters’ clinics between 2010 and 2015.

### 2.1 Study population

Women were eligible for inclusion if they were aged ≥18 years, working at the study site and reported exchanging sex for money in the past 30 days.

### 2.2 Intervention setting

The study was conducted in Mutare, a small city bordering Mozambique; Victoria Falls, a tourist town bordering Zambia; and Hwange, the location of a large colliery. The 2011 survey was conducted eight months after initiation of the intensified community mobilization, while the 2015 round took place 54 months after implementation.

### 2.3 The intervention

In 2010, we intensified community mobilization in Mutare, Hwange and Victoria Falls, providing peer‐delivered activities biweekly instead of monthly. We hired additional peer educators, who brought FSWs together in participatory workshops to build solidarity and reduce competition. We further trained public sector health workers to be more “sex worker friendly” through a three‐day workshop, and five‐day attachment allowing them to shadow nurses at the Sisters’ clinics, and monthly meetings with Sisters clinic staff and peer educators. The aim of intensified community mobilization was to foster an enabling environment for sex workers to adopt protective behaviours and increase their use of health services. In 2014, specific activities designed to engage younger women were introduced 34.

### 2.4 Sampling and recruitment for RDS survey

In the absence of a sampling frame, we used Respondent Driven Sampling (RDS) to recruit women. While there is debate on the extent to which RDS achieves representativeness 35, 36 it is a recommended approach for recruiting hidden populations 37 and has been used by other recent studies among FSWs in southern Africa 37, 38.

First, we conducted rapid ethnographic mapping to inform the feasibility and design of the RDS survey, including selection of initial recruiters (seeds) 38. Seeds were purposively selected to represent the range of FSWs’ ages, geographic areas and sex‐work typologies identified during mapping. Sample sizes reflected site size, mapping findings, and number of women seen in the programme to date. Ten seeds were recruited from Mutare and six each from Hwange and Victoria Falls, which are smaller towns. Each seed completed a questionnaire, provided a finger prick blood sample for HIV antibody testing, and was issued with two uniquely identified coupons. Seeds passed these coupons on to FSWs who met study inclusion criteria, inviting them to the study. On presenting a coupon, FSWs were assessed for eligibility and asked to provide written consent to participate. Each recruit was given a further two coupons for peer referral. Participants received US$5 to cover costs of participation, with an opportunity to earn US$2 for each successfully recruited referral.

### 2.5 RDS survey data collection

A questionnaire was developed in English, translated into Shona and Ndebele, and pilot tested. Responses were entered directly into a computer‐assisted survey instrument (QDS™ Nova research Company in 2011 and Open Data Kit in 2015). Data were collected anonymously, covering socio‐demographic and economic variables, sexual behaviour, mental and physical health, history of STIs, sexual and social networks, social capital, utilization of services including HIV testing, ART, PMTCT and family planning.

### 2.6 Laboratory procedures

Finger prick blood samples were collected onto filter paper by a nurse and transported to the National Microbiology Reference laboratory in Harare. They were tested for HIV‐1 in series using AniLabsystems EIA kit (AniLabsystems Ltd, Fin‐01720, Vantaa, Finland) with specimens testing positive re‐tested using Vironostika^®^ HIV Microelisa Systems BioMerieux, Inc, Durham NC 27704. Discrepant results were resolved using Western blot. Referral for HIV testing was made freely available in the Sisters clinics.

### 2.7 Qualitative data collection and analysis

We conducted semi‐structured interviews in 2015 with all study seeds (10 FSWs in Mutare, six in Hwange, and six in Victoria Falls). Interviews explored FSWs’ perceptions of their work, access to and quality of health services, and their experiences of community networks and support. All interviews were conducted in Shona or Ndebele. Audio‐recorded data were transcribed and translated verbatim into English, and entered into NVivo 8 (QSR International Ltd, Melbourne, Australia) for coding. For this paper, we extracted data referring to knowledge, participation, and perceptions of the intensified community mobilization.

Following data familiarization, the third author conducted “broad brush” thematic coding on perceptions of available health services, use of services, and facilitators and barriers to accessing both public service and targeted Sisters clinics; this was followed by more detailed inductive coding. Analysis of qualitative data was conducted specifically to complement and help explain quantitative findings on differences over time in uptake of Sisters clinical and community services, in relation to the “intensified” components, and in comparison to other available healthcare.

### 2.8 Programme data review

Programme data for 2010 to 2015 was drawn from the Sisters’ data collection system which collects client data electronically in real time. Nurses enter data into the system using a tablet at each client visit. A unique identifier is allocated and demographic data are recorded during the first visit. At the first and all subsequent visits clinical data including presenting problems, services offered and diagnoses are collected.

### 2.9 Key indicators

Key indicators assessed were drawn from the RDS surveys, namely: HIV prevalence, knowledge of HIV status among HIV‐positive FSWs and testing in the last 6 months amongst HIV‐negative FSWs, engagement with care and ART use among HIV‐positive FSWs, and reported condom use with clients and regular partners. We also investigated self‐reported service uptake including contact with peer educators, visits to the clinic, testing behaviour and perceived social cohesion.

We classified individuals who were HIV positive as knowing their status if they tested positive on the survey and self‐reported that their last HIV test was positive, and on ART if this was self‐reported, or HIV‐negative if they tested negative for HIV and reported that they had tested negative within the last 6 months.

### 2.10 Statistical analysis

We describe the prevalence of programme engagement and key HIV status, prevention and care indicators in 2011 and 2015, accounting for the RDS design using RDS‐I weighting 39 to be consistent across years, as previously reported for the 2011 survey 33. For 2015, we used the R RDS package version 0.7 to 8 40, setting identical options to those used in the Stata RDS package 41 in 2011. Bootstrapping using the Salganik 2006 method was used to obtain the confidence intervals 42, 43 approximating bootstrapped estimates to the t distribution. The number of bootstrap samples used to generate the confidence intervals were those necessary to compute the standard error to accuracy 0.001.

While we cannot attribute changes in HIV prevention and care indicators to the Sisters programme, we are interested in assessing whether differences in indicators between 2011 and 2015 suggest improvements in engagement with HIV prevention and care services. To determine evidence of bias in RDS estimates at each time‐point due to non‐convergence of sample waves (dependence on seed characteristics) we assessed the cumulative estimate over sample waves (Appendix 1) 44. We also checked whether an observed difference in key indicator prevalence between 2011 and 2015 could be due to differences in the demographic composition of sex workers present at the sites. To do this, we pooled data across years for each site and used logistic regression models to examine whether there was evidence for an effect of year on the outcome of interest, adjusted for age, duration in sex work and education (same categories as used in the descriptive analyses, not assuming linearity). For these models, we dropped the seeds and weighted data by the inverse of reported network size, normalized by year so changes in differences in network size by year would not affect results. This approach is in line with other regression analyses using RDS data 45, 46.

Programme level data over the course of the intervention was collated by site showing trends in clinic visits and other key indicators by year.

### 2.11 Ethical approval

All participants gave written informed consent collected according to the principles of Good Clinical Practice. Approval for the study was given by the Medical Research Council of Zimbabwe (MRCZ), the UCL Ethics Committee, and the London School of Hygiene and Tropical Medicine Ethics Committee.

FSWs were consulted on intervention design and peer educators were actively involved throughout its implementation.

## 3 Results

### 3.1 RDS recruitment

In 2015, 913 women were included in analysis: 407 in Mutare, 255 in Hwange and 251 in Victoria Falls compared to 836 women in 2011: 370 in Mutare, 237 in Hwange and 229 in Victoria Falls. Following the seeds, there were five additional recruitment waves in all sites in 2011 and six in 2015. Appendix 1 shows estimated convergence over sample waves in each year, which include initial seed characteristics, also previously published 33.

### 3.2 Characteristics of sampled population

FSWs’ median age in both years was 33. The age distribution remained similar over time in Mutare and Hwange, however the proportion of those younger than 25 years changed from 39.5% (95% CI 30.3 to 48.7) in 2011 to 16.3% (95% CI 12.1 to 20.5) in 2015 in Hwange.

More women reported no education or incomplete primary education in 2011 than 2015 (Table 1). Duration in sex work was shorter in 2015 than in 2011 in Mutare, with more women reporting fewer than five years in sex work. In all sites, across years, the majority of participants reported finding clients in bars or nightclubs. Duration at site did not change for those who had stayed at the site for up to 40 years however for more than 40 years was higher in Mutare and Victoria Falls in 2015.

**Table 1:** Sociodemographic characteristics of female sex workers in Hwange, Mutare and Victoria Falls

	Hwange								Mutare								Victoria Falls							
	2011				2015				2011				2015				2011				2015			
	n = 237	%	95% CI		n = 255	%	95% CI		n = 370	%	95% CI		n = 407	%	95% CI		n = 229	%	95% CI		n = 251	%	95% CI	
Education																								
None or incomplete primary	21	12.7	7.1	18.3	3	1.2	−0.4	3.1	49	12.9	8.4	17.4	9	2.2	0.6	4.8	34	15.5	9.1	21.9	8	3.2	1.1	6.2
Incomplete secondary	118	55.3	46.9	63.8	137	53.9	52.9	66.8	186	49.7	42.8	56.6	264	65.2	58.7	69.7	146	67.5	59.1	75.9	170	67.7	63.2	75.6
Complete secondary or higher	87	32.0	24.1	39.9	114	44.9	31.9	45.6	134	37.3	30.4	44.2	132	32.6	27.7	38.5	49	17.0	10.3	23.7	73	29.1	21.0	32.9
Duration in sex work																								
Up to 2 years	38	16.0	17.9	11.7	51	26.1	17.5	34.7	42	11.4	10.1	7	81	22.0	17.0	27.0	59	25.8	31.5	22.7	70	30.7	23.1	38.2
2 to 5 years	87	36.7	37.9	30.6	68	31.1	23.0	39.2	130	35.1	40.6	33.9	86	22.8	17.5	28.1	100	43.7	39.2	32	68	27.9	21.4	34.4
More than 5 years	112	47.3	44.2	36.9	136	42.8	35.0	50.6	198	53.5	49.4	43.1	240	55.2	49.4	61.1	70	30.6	29.3	22.5	113	41.4	34.5	48.3
Duration at site																								
<5 years	57	25.0	17.5	32.6	59	26.4	19.0	33.8	60	20.7	14.3	27.1	65	17.7	13.1	22.3	74	40.1	30.5	49.7	66	29.1	22.0	36.2
5 to 10 years	28	16.0	8.7	23.3	37	17.2	10.2	24.2	53	14.0	9.4	18.6	48	10.5	7.5	13.4	52	21.8	14.1	29.6	56	27.5	19.0	35.9
10 to 20 years	32	14.9	7.7	22.2	40	15.2	10.4	19.9	66	14.7	9.8	19.6	76	20.2	15.0	25.5	57	20.0	13.0	27.0	61	19.6	15.0	24.2
20 to 40 years	97	36.3	27.8	44.7	95	32.0	25.8	38.2	153	43.4	35.7	50.8	157	36.3	31.1	41.5	46	18.1	11.7	24.4	60	20.2	15.0	25.3
>40 years	23	7.7	3.3	12.2	24	9.2	5.5	12.8	38	7.3	3.8	10.8	60	15.3	11.2	19.4	0	0.0	0.0	0.0	8	3.7	1.3	6.0
Where find clients^a^																								
Bars/night clubs/entertainment	192	80.5	74.2	86.8	173	69.2	63.0	75.5	298	80.7	75.5	85.9	327	81.9	75.9	87.8	217	94.5	90.7	98.3	193	73.4	65.3	81.6
Telephone/at home	89	38.9	30.9	46.9	7	2.7	0.9	4.5	106	26.8	20.7	32.9	16	3.1	1.6	4.6	42	15.8	9.0	22.6	5	1.6	0.6	2.6
Market stalls	6	3.5	0.4	6.6	12	5.2	2.2	8.3	32	8.2	4.8	11.6	52	12.9	9.4	16.5	2	0.4	−0.2	1.0	13	5.5	1.5	9.4
On the street	71	26.0	18.7	33.3	0	0.0	0.0	0.0	133	35.5	29.4	41.6	3	0.7	−0.3	1.7	30	9.8	5.7	13.9	1	0.3	0.3	0.4
Lodges	18	5.0	2.4	7.7	63	22.8	17.0	28.5	30	5.9	3.1	8.7	5	0.8	0.3	1.3	12	2.0	0.4	3.6	38	19.2	11.2	27.2
Hotels	11	2.4	0.7	4.2	0	0.0	0.0	0.0	19	6.2	3.1	9.3	1	0.7	−4.9	6.2	14	3.4	1.0	5.8	0	0.0	0.0	0.0
Other	20	7.3	3.8	10.9	0	0.0	0.0	0.0	32	7.5	4.4	10.6	0	0.0	0.0	0.0	6	1.7	−1.2	4.6	0	0.0	0.0	0.0
Where collected condoms in the last year																								
Grocery store	37	16.0	10.1	21.8	17	5.2	2.6	7.7	143	34.6	29.4	39.9	58	16.4	11.0	21.8	50	23.5	16.4	30.7	24	13.4	5.0	21.7
Peer educators	17	7.5	3.7	11.2	8	1.8	1.0	2.6	23	4.9	3.3	6.4	15	4.0	1.0	7.0	17	8.4	4.2	12.5	10	3.3	1.3	5.2
Local clinic/hospital	114	50.6	43.2	58.0	104	43.5	36.1	50.9	164	45.7	39.8	51.6	182	46.7	41.0	52.4	93	39.3	32.2	46.4	130	53.1	46.1	60.1
Sisters’ clinic	23	9.4	6.0	12.8	178	62.9	54.2	71.7	24	5.9	3.9	7.9	235	52.7	46.7	58.8	33	10.8	7.8	13.9	130	47.8	40.6	55.0
Bars	63	24.9	19.4	30.5	40	17.0	11.4	22.6	105	30.3	24.7	35.9	19	3.8	1.8	5.8	85	38.7	31.4	46.0	36	13.1	8.5	17.6

a These figures are not directly comparable over time. In 2011 participants could list more than one location, whereas in 2015 they could choose only one.

Where FSWs reported they found clients was not comparable because in 2011 participants could list more than one location, whereas in 2015 they could choose only one.

There were significant changes in places where FSWs reported they collected condoms with exception of local clinic or hospital which did not change. FSWs who reported collecting condoms from bars and from peer educators reduced in Hwange and Mutare, but did not change in Victoria Falls. Reported condom collection from Sisters clinics increased between 2011 and 2015 across all three sites.

### 3.3 Sisters programme engagement 2011 and 2015

In 2011, 18.8% (95% CI 15.1% to 22.6%), 31.6% (95% CI 26.4% to 36.7%), 29.8% (95% CI 23.7% to 35.9%) FSWs reported having visited the Sisters clinic in the last 12 months in Hwange, Mutare and Victoria Falls respectively. In 2015, the time period over which clinic attendance was assessed was reduced to the previous 6 months and was 77.9% (95% CI 69.1% to 86.7%), 47.0% (95% CI 40.9% to 53.2%) and 50.1% (95% CI 42.4% to 57.9%) in Hwange, Mutare and Victoria Falls respectively.

Contact with a peer educator in the last 12 months was reported by 32.9% (95% CI 26.6% to 39.1%), 31.1% (95% CI 27.8% to 38.4%) and 36.9% (95% CI 29.9% to 43.8%) of women in 2011. In 2015 the time period for contact with peer educators was changed from last 12 to last 6 months, and the proportion reporting contact was perhaps as a consequence reduced to 30.3% (95% CI 24.5% to 36.1%), 20.4% (95% CI 16.7% to 24.0%) and 26.1% (95% CI 21.0% to 31.2%) in Hwange, Mutare and Victoria Falls. The proportion of women who reported receiving condoms from the Sisters clinic was higher in 2015 than in 2011 in all sites. A lower proportion of women reported receiving condoms from peer educators in Hwange (Table 1).

### 3.4 HIV status, prevention and treatment

**Figure 1:**
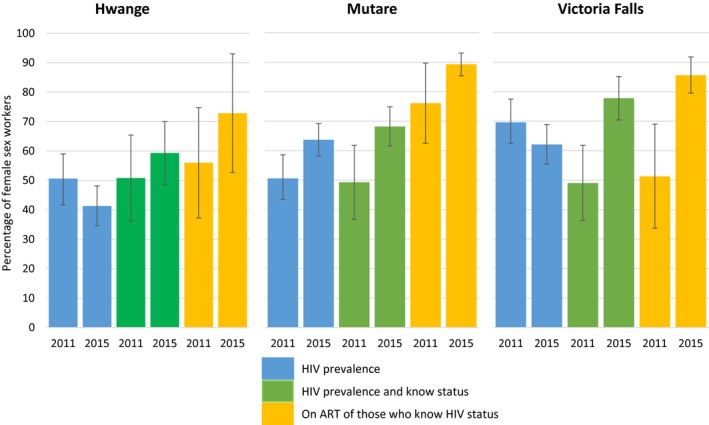
Comparison between 2011 and 2015 of HIV prevalence, knowledge of positive status, and antiretroviral therapy (ART) among female sex workers in Hwange, Mutare and Victoria Falls.

HIV prevalence in Hwange was higher in 2011 than 2015 41.3% (95% CI 34.6 to 48.1), a pattern similar in Victoria Falls with 69.6% (95% CI 61.7 to 76.7) HIV‐positive in 2011 and 62.1% (95% CI 55.3 to 68.7) in 2015. In Mutare, HIV prevalence was observed to be higher at 63.7% (95% CI 58.2% to 69.2%) in 2015, to 50.6% (95% CI 43.5% to 58.6%) in 2011 (Figure 1). After adjusting for sociodemographic differences, we observed reduced odds of being HIV‐positive amongst FSWs in Victoria Falls (aOR = 0.53, *p* = 0.022) in 2015 compared to 2011 in Hwange (aOR = 0.54, *p* = 0.031). Evidence of an increase in prevalence in Mutare reduced significantly once adjusted (aOR = 1.45, *p* = 0.085) (Table 2).

**Table 2:** Differences in HIV status, knowledge of status and whether on antiretroviral therapy (ART) between 2011 and 2015

	Crude odds ratio	95% CI		Wald test *p* value	Adjusted OR	95% CI		Wald test *p* value
**Victoria falls**								
HIV positive								
2011	1				1			
2015	0.69	0.42	1.14	0.147	0.53	0.31	0.91	0.022
Knowledge of status among those HIV positive								
2011	1				1			
2015	2.83	1.46	5.49	0.002	2.26	1.06	4.83	0.035
On ART among those HIV positive and aware of their status								
2011	1				1			
2015	5.22	2.19	12.44	<0.001	4.38	1.73	11.05	0.002
**Mutare**								
HIV positive								
2011	1				1			
2015	1.62	1.11	2.39	0.014	1.45	0.95	2.2	0.085
Knowledge of status among those HIV positive								
2011	1				1			
2015	2.18	1.3	3.65	0.003	2.76	1.53	4.96	<0.001
On ART among those HIV positive and aware of their status								
2011	1				1			
2015	2.72	1.2	6.16	0.018	3.63	1.14	11.59	0.03
**Hwange**								
HIV positive								
2011	1				1			
2015	0.69	0.43	1.1	0.122	0.54	0.31	0.94	0.031
Knowledge of status among those HIV positive								
2011	1				1			
2015	0.99	0.53	1.84	0.963	1.03	0.54	1.98	0.925
On ART among those HIV positive and aware of their status								
2011	1				1			
2015	2.85	1.12	7.26	0.03	1.21	0.41	3.59	0.729

There was evidence of an increase in the odds that HIV‐positive women knew their status in Victoria Falls (aOR = 2.26 *p* =0.035) and Mutare (aOR = 2.76, *p* < 0.001), but not in Hwange.

There was strong evidence that HIV‐positive FSWs who reported being aware of their status were more likely to report being on ART in 2015 in Victoria Falls (aOR = 4.38, *p* = 0.035) and in Mutare (aOR = 3.63, *p* = 1.14 to 11.59). While a similar, though smaller, effect was seen in Hwange in the unadjusted results, there was no longer evidence for this effect (aOR = 1.21, *p* = 0.729) after adjustment.

**Figure 2:**
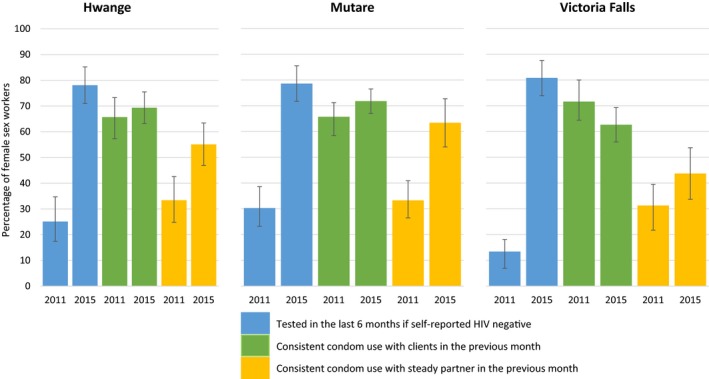
Comparison between 2011 and 2015 in proportions of women testing HIV negative and reporting recent HIV testing and condom use in Hwange, Mutare and Victoria Falls.

There was strong evidence that the proportion of HIV‐negative FSWs who reported testing for HIV in the previous 6 months was much higher in 2015 compared to 2011. In Hwange, it increased from 25.1% (95% CI 17.4% to 34.7%) to 78.1% (95% CI 71.0 to 85.2), in Mutare from 30.3% (95% CI 23.2% to 38.6%) to 78.6% (95% CI 71.7% to 81.5%), and in Victoria Falls from 13.4% (95% CI 8.7% to 19.9%) to 80.8% (95% CI 74.0 to 87.7). This effect remained even after adjustment (Figure 2).

There was little evidence of a difference in consistent condom use with clients in the past month in 2011 and 2015, except in Mutare, where an increase was observed in 2015 after adjustment (aOR = 1.50, *p* = 0.058).

In Mutare and Hwange, condom use with regular partners was higher in 2015 compared to 2011, (aOR = 2.44, *p* = 0.003 and aOR = 2.51, *p* = 0.003), but there was little evidence of a difference in Victoria Falls.

### 3.5 Qualitative results

Interviews conducted with the 22 RDS seeds in 2015 illustrate how FSWs perceived the intensified community mobilization. All 22 respondents reported knowing about the local Sisters clinic, and having used it at least once, although this was not a prerequisite for study recruitment. Table 3 presents excerpts from FSWs’ accounts of why they engaged with Sisters, how they felt sex workers’ health and wellbeing were affected over the intervention period, and reasons not all FSWs were reached through the programme.

**Table 3:** FSW perceptions of intensified service

Services	Excerpts from interviews with seeds selected as RDS recruiters in 2015 about *Sisters with a Voice* services
Quality of care	The services at Sisters with a Voice clinic are of quality, especially on drugs. They are not shady when it comes to drugs. If the course is for seven days, they will give you exactly seven, but at other clinics they will tell you that the course was supposed to be for seven days but due to the shortage of pills ‘we are now giving you four.’ (Hwange, 44, sex worker for 10 years, bars) Here at the sister's clinic there is privacy, if I have a wound ‘down there’ I can come here and say ‘sister, I am not sure about what is happening, the condom burst so now look at the wound I have’, but at the hospital they will be saying ‘go away, we are busy.’ (Victoria Falls, 37, sex worker for 13 years, home)
Facilitators of service use	I chose to come to this clinic because I get treatment for free and the medication that I want is available. If I go to [other clinic] they can tell me to pay 2 dollars. … They will tell me to buy medication after paying 2 dollars or they will say they don't have it. … And [at Sisters] they treat us well, they don't ask me where I contracted the STI, they don't care about that, they just treat us like people (Mutare, 33, sex worker for 15 years, bars & truck stops) You feel free. We used to cry, you know when you get to a queue and you are asked, ‘how are you this morning?’ We didn't experience this when we were growing up, a person saying good morning to you girls, smiling. … We want our nurses to welcome us and just say ‘good morning’. It has an impact. … That is what we liked at Sisters with a Voice clinic (Mutare, 47, sex worker for 33 years, bars) I first came to attend meetings, I came to learn and see how it goes. Then I got sick and they helped me, so then I said ‘I can get help here and also some lessons.’ … Yes it has helped me because I managed to tell others and they are now able to come. I think I can say that, this is our place. … I am comfortable about this place (Victoria Falls, 23, sex worker for 5 years, bars)
Barriers to service use	Ah, I can say that these are the type of sex workers who will not want it to be known, they do not want to show that they are sex workers. … Yes, they know that this clinic is for sex workers, so maybe this person will be pretending that they are good [not selling sex], so for her to be seen at a sex workers’ clinic would not be good because other people will be asking why she is going there. (Hwange, 38, sex worker for 2 years, bar based) … some people tell themselves that if they go then ‘others will see what I do’ so they don't want to be seen, they hide themselves (Victoria Falls, 23, sex worker for 5 years, bars) The way we [sex workers] think is different [from each other]. There is a person to whom I said ‘here is a voucher, go to the clinic’ and she said ‘ah I don't have the time.’ So she might think that it is not important. … Yes, they are those who say ‘we will never set a foot there’. (Mutare, 27, sex worker for 4 years, nightclub)
Community outreach	When I started attending Sisters meetings, we were taught by mobilization ‐ they used to touch on everything, the way we take care of children, how we take medication, and taking care of ourselves, and that even if I am a sex worker I should love myself as I am. (Mutare, 33, sex worker for 15 years, bars & truck stops) It's very important to have peer educators. … I didn't know that there was a clinic, but if they move around our houses or bars and find people and tell them to come to the clinic if they are sick, there will be others who are sick and don't know where to go but if they get people who advise them to go to such a place they can come and get help. (Victoria Falls, 29, sex worker for 3 years, bars) Ah peer educators say ‘girls we are wanted at work, take a bath and let's go’. They wait for us. … ‘Girls here are some condoms’. They carry them in their bags. ‘Girls, here are some condoms, take some condoms. Here are some condoms.’ Our peer educators have love. (Mutare, 20, sex worker for 8 years, bars & street) What has changed is that sex workers are more united because isn't you see that some of us meet here, it adds, it's a bond. We won't ignore each other when we meet. We greet each other and also we know each other better that that one is a sex worker. (Mutare, 34, sex worker for 8 years, bars)
Change over time	It has changed people's lives, because people are now being tested, they didn't want to be tested at the hospital … but here at Sisters clinic they come. (Victoria Falls, 29, sex worker for 3 years, bars) Ah, I think that it has made a huge difference … I can say that most of us would just get unplanned pregnancies, you would [often] see a sex worker pregnant, so they [Sisters] are helping them a lot because they offer depo and other family planning tablets, and they give us condoms so that we do not get sexually transmitted infections (Hwange, 38, sex worker for 2 years, bar based) Yes their health, and it has reduced the rate of STIs because they give us condoms for free, lately I have not heard that there is a sex worker who is seriously ill from an STI. … Even the rate of getting pregnant has reduced. (Victoria Falls, 24, sex worker for 2 years, bars) When the clinic started in 2010, or was it 2011, there were just a few people who would come because they were scared. But now there are many, and they even come to ask when the [mobile] clinic will be coming because they want to go there, something which never used to happen back then, but now a lot of people come to the clinic, the attendance has increased now (Hwange, 31, sex worker for 8 years, street & highways)

Respondents overwhelmingly praised the clinics, which were perceived to be the mainstay of the Sisters programme. Respondents compared Sisters favourably to other public or private facilities where user fees were charged, staff were considered insensitive and sometimes discriminatory and hostile towards FSWs. FSWs highlighted the welcoming attitude of nurses, free services, comprehensive vaginal examinations, and reliable supply of medication as key advantages of the Sisters clinic.

FSWs also described how the community's trust in Sisters took time to develop. Several reflected back to when the local Sisters clinic was first established, describing widespread fears that they might be “outed” as sex workers. Introduction of peer educators and community mobilization meetings was seen to streamline the trust‐building process and increase the rate at which FSWs were willing to attend the clinical services. Some women described coming for meetings first and then felt confident to attend the clinic.

The interactive sessions were also credited with expanding FSWs’ awareness of self‐care and prevention and treatment. Regular HIV testing was particularly seen as a proactive measure FSWs now took. Most interviews mentioned positive changes in relations between FSWs, suggesting that the community mobilization process led to feeling more “united” and willing to cooperate and help one another, particularly in encouraging each other to seek treatment at the clinic and get HIV tested.

However, respondents also described barriers to engaging with Sisters services. Not all sex workers could be persuaded to attend, particularly sex workers who wanted to hide their involvement in sex work. Others did not prioritize health, and were described as “lazy” “ignorant” or simply “thinking differently” compared to FSWs who had taken up the targeted services.

### 3.6 Programme review results

Increases were observed in clinic visits across all sites, with the highest increase in Mutare, from 65 FSWs in 2010 to 1514 FSWs in 2015. The number of new FSWs attending clinics also increased. The lowest increase was observed in Hwange from 65 new FSW attendees in 2011 to 171 new FSW attendees in 2015. The number of FSWs’ testing and diagnosed with HIV increased in all sites; the greatest increase in Mutare, where 25 FSWs were tested in 2010 compared to 518 FSWs in 2015 (Table 4).

**Table 4:** Clinic attendance and clinical services received by female sex workers (FSWs) between 2010 and 2015

	2010	2011	2012	2013	2014	2015
**Hwange**						
Number of total SW reached with clinics	65	267	598	962	811	986
Number of new SW reached with clinics	65	132	235	267	181	171
SW tested for HIV at the sites	7	23	59	139	81	148
SW who knew their HIV status at first visit	42	101	192	233	167	160
**Mutare**						
Number of total SW reached with clinics	65	713	1,093	782	1,084	1,514
Number of new SW reached with clinics	52	523	461	239	468	556
SW tested for HIV at the sites	6	93	199	186	63	426
SW who knew their HIV status at first visit	25	394	412	211	430	518
**Vic falls**						
Number of total SW reached with clinics	74	341	275	257	353	630
Number of new SW reached with clinics	74	154	66	55	136	197
SW tested for HIV at the sites	6	41	22	15	38	92
SW who knew their HIV status at first visit	47	112	57	47	121	189

## 4 Discussion

This paper compares results from RDS surveys conducted in 2011, eight months after the implementation of intensified community mobilization, and in 2015, after 54 months in three sites. We also show programme reach between 2010 and 2015 and capture FSWs’ feelings and perceptions about the programme. There was some evidence of change in HIV prevalence, knowledge of status and linkage to ART, and condom use with regular partners. Sisters’ clinics reached more women by 2015. Qualitative data suggests improved familiarity with the programme, growing trust in its intentions, and regular opportunities to engage with each other during peer‐led community meetings might have been the mechanisms through which the programme increased FSWs’ engagement with care.

We used RDS to obtain estimates as representative as possible of the FSW population at each site. In assessing differences between 2011 and 2015, we used very similar protocols and reviewed RDS performance. We examined our quantitative results alongside qualitative data demonstrating FSWs’ observations regarding service use, as well as clinic records showing FSW engagement with Sisters clinics.

Consistent with previous studies, we found low condom use with regular partners in 2011 47, 48. This study suggests that peer education and intensive community outreach and participation can improve condom use with regular partners 49. Similar positive impact was observed for clinic attendance. While receiving condoms from a peer educator did not show any change, this may have been compensated by FSWs’ collecting condoms directly from clinics, which increased across sites.

HIV prevalence among FSWs remains three to four times higher than that of women aged 15 to 49 in Zimbabwe's general female population, which was 17.7% 50 in 2011 and 16.6% in 2015 51. This is consistent with other studies showing HIV prevalence as high as 50% to 60% among FSWs 46, 52. Observed changes in HIV prevalence, while adjusted for socio‐demographic differences of the samples in 2011 and 2015, could have been affected by changes in incidence and in the proportion successfully initiated and sustained on treatment, and subject to unmeasured confounding. HIV prevalence in the general population did not change between 2011 50 and 2015 51. The proportion of FSWs aware of their HIV‐positive status and, among those who were on ART, was significantly higher in Victoria Falls and Mutare in 2015 than in 2011, even after adjustment.

These findings are similar to previous research among FSWs in Zimbabwe 53, although the proportion on ART was higher than the estimated 38% to 39.3% 54, 55 in other settings. The Sisters’ clinic in Hwange was available twice a week at an outreach site, compared to daily in Victoria Falls and Mutare at static clinics, which may explain variations. The percentage of FSWs taking ART of those who know their status in the three sites is similar to that of women in the general population at 87.3% 51. However, because there remain gaps in knowledge of status, the percentage of all HIV‐positive FSWs on ART falls short of the 90‐90‐90 target, which would mean 81% of all FSWs living with HIV on treatment. In fact, 39.5% in Hwange, 61.5% in Mutare and 66.8% in Victoria Falls of all HIV‐positive FSWs reported taking ART in 2015. We did not measure viral suppression, but another study in Zimbabwe showed viral suppression between 67% and 72% among HIV‐positive FSWs 53. As testing coverage increases, innovative solutions will be required to reach women who have not yet tested, or who test infrequently. If viral suppression is high among FSWs, further transmission into the general population will reduce.

HIV testing is a critical entry point for HIV services 56. We detected large increases in HIV‐negative FSWs testing in the last six months, six times higher in Victoria Falls. This meets WHO recommendations for FSWs to test annually 57. Studies from Kenya have shown increases in testing among FSWs where friendly and targeted services are offered 58. Uptake of preventive behaviours such as reported condom use with clients did not improve as anticipated by the intensified services. Programmes will need to better link HIV‐negative FSWs to prevention services including PrEP as part of a combination prevention strategy 59, 60.

We suggest that the FSWs’ positive perception of the Sisters programme helped overcome barriers to testing 61, 62, and peer‐led interactions with other HIV‐positive FSWs reduced anxiety associated with receiving a positive result. However, we did not interview anyone who claimed to face barriers to attending the Sisters clinics, therefore were unable to examine which aspects of the programme were less welcoming and accessible, and who felt excluded as a result. Furthermore, FSWs compared Sisters services favourably to other local facilities, suggesting that the training component of the intervention, which aimed to reduce discrimination by public sector health workers and make them more “sex worker friendly,” did not demonstrably change FSWs’ views of these services.

Community mobilization sessions were seen as a good opportunity to unite FSWs and increase peer support for accessing HIV services. This resonates with previous findings that strong peer support networks are associated with willingness to engage with testing, care, treatment initiation and adherence 63, 64, 65. There is a need to learn from other contexts how to build strong FSW movements with effective solidarity and social cohesion. Positive effects of strong sex worker networks have been observed, particularly in Asia 66, 67.

The study design had some limitations. It was not possible to enrol a control arm, making it difficult to attribute changes observed to the intervention. Changes in engagement in care for HIV‐positive FSWs may have been confounded by developments in the National ART programme, which expanded during the intervention period. Demographics of study participants for the two surveys were different and may have influenced key indicators, for example, HIV prevalence. The first survey was conducted eight months into implementation, ideally this should have been done prior to the intervention's commencement, to allow for a better estimation of a baseline.

## 5 Conclusion

Improvements in key HIV care engagement indicators were observed among FSWs in two sites and in testing and prevention indicators across the three sites after implementation of an intensified community mobilization intervention. The experiences of community mobilization programmes targeting key populations including sex workers are critical to inform scale up and translation into sustainable policies and programmes. Integrated, peer‐led approaches to biomedical interventions for FSWs that include ART and PrEP may help countries achieve the 90.90.90 targets by 2020.

## Competing interests

The authors declare that they have no competing interests.

## Authors’ contributions

FMC, SM, JD and SM conceived and designed the experiment. TNM, EF, CD, XA, SM, JB and JRH analysed the data. TNM and EF wrote the manuscript. FMC, JB, JRH, CD and XA assisted in drafting the manuscript.

## Funding

The 2011 and 2015 data collection was funded by The Deutsche Gesellschaft für Internationale Zusammenarbeit GmbH (GIZ).

## Supporting information


**Appendix 1.** RDS Diagnostics in 2011 and 2015.
**Appendix 2.** Respondent Driven Sampling Questionnaire 2015 in word format.
