# Supplementary material for: Changes in engagement in HIV prevention and care services among female sex workers during intensified community mobilization in 3 sites in Zimbabwe, 2011 to 2015
Source: J Int AIDS Soc. 2018 Jul 22;21(Suppl Suppl 5):e25138. doi: 10.1002/jia2.25138 (PMC6055130; doi:10.1002/jia2.25138)
Supplement: Supplementary file 1 — Appendix 1. RDS Diagnostics in 2011 and 2015. Appendix 2. Respondent Driven Sampling Questionnaire 2015 in word format. [file JIA2-21-e25138-s001.docx]

Appendix RDS Diagnostics in 2011 and 2015

The weighting procedure that allows RDS surveys to serve as an approximation of a simple random sample assumes that enough sampling waves have occurred such that the characteristics of the sample no longer depend upon the characteristics of the seed participants. To assess this, a plot showing the change in the weighted estimate over cumulative sample size have been recommended. The sample is judged to have ‘converged’ when it has remained relatively stable. If it is still rising, it is possible that it would have continued to do so had the sample size been larger and correspondingly, that the RDS-weighted estimate from the survey is either an over or under-estimate. This possible bias is particularly important when assessing the evidence for a change between two RDS surveys over time, as in our analysis.

Here, we assess the convergence for key HIV care cascade outcomes in the RDS surveys from 2011 and 2015 in each site and the potential for likely over or underestimation of the true proportion.

Interpretation.

Convergence of the HIV estimates appeared reasonable in 2011. In 2015, the estimates in Mutare and Hwange might be somewhat over-estimated, Figure 1.

In Mutare in 2011, the proportion of women who were HIV posititve and aware of their status does not appear to have converged so might have been under-estimated, Figure 2. In 2015, all site estimates appeared to have converged.

The estimate for the proportion of women who were HIV positive, knew their status and on ART might have continued to rise in Hwange with a higher sample size and might therefore be an under-estimate, Figure 3. Convergence in 2015 appears reasonable in Hwange, but might have continued to rise in Mutare and Victoria Falls, where we might be under-estimating the true proportion.

**Figure 1 HIV prevalence: RDS-weighted proportion of women testing HIV positive as sample accumulates**

Hwange 2011 Hwange 2015

Mutare 2011 Mutare 2015

Victoria Falls 2011 Victoria Falls 2015

**Figure 2: HIV positive and aware of status: RDS-weighted proportion of women testing HIV positive and reporting that they had previously tested positive as sample accumulates**

Hwange 2011 Hwange 2015

Mutare 2011 Hwange 2015

Victoria Falls 2011 Victoria Falls 2015

**Figure 3: HIV positive, aware of status and on ART: RDS-weighted proportion of women testing HIV positive and reporting that they had previously tested positive and were on ART as sample accumulates**

Hwange 2011 Hwange 2015

Mutare 2011 Hwange 2015

Victoria Falls 2011 Victoria Falls 2015
